# Soil Organic Matter Composition and pH as Factors Affecting Retention of Carbaryl, Carbofuran and Metolachlor in Soil

**DOI:** 10.3390/molecules28145552

**Published:** 2023-07-20

**Authors:** Irmina Ćwieląg-Piasecka

**Affiliations:** Institute of Soil Science, Plant Nutrition and Environmental Protection, Wroclaw University of Environmental and Life Sciences, Grunwaldzka 53 St., 50-357 Wrocław, Poland; irmina.cwielag-piasecka@upwr.edu.pl; Tel.: +48-71-320-5635

**Keywords:** pesticides, soil organic matter, organic carbon fractions, sorption, pH

## Abstract

The majority of studies concerning the environmental behavior of hydrophobic pollutants in soil consider soil organic matter (SOM) content as a main factor influencing chemical retention, whereas the composition of SOM and its individual fraction share are often neglected. In the present paper, carbaryl, carbofuran and metolachlor retention by loamy sand and loam topsoil materials is compared and referred to humic acids (CHA) and the residual carbon (CR) content of SOM. Additionally, the sorption-desorption behavior of agrochemicals in soils was tested at a pH of three to seven. Calculated isothermal parameters point to favorable, spontaneous and physical pesticide sorption. Groundwater ubiquity score (GUS) indexes confirmed the low leaching ability of metolachlor on soils and moderate of carbofuran. The high affinity of carbaryl to CR may explain its pronounced sorption in loam soil and the lowest percolation potential. Carbofuran retention in soils was associated with montmorillonite (Mt) and CR fractions. Meanwhile, metolachlor uptake was related to humic acid and Mt content of the soils. Lower pH enhanced retention of the agrochemicals, except for carbaryl sorption in sandy loam soil. Results of this study highlight that SOM composition and mutual share of individual organic carbon fractions alongside pH may play a crucial role in predicting non-ionic pesticide behavior in soil.

## 1. Introduction

The effect of soil composition on pesticide sorption has been widely studied since these substances were introduced into the environment [1,2,3,4]. There are many pedological factors governing their retention. Among them, native organic matter content [4,5] as well as the presence of exogenous sources of OM (biochar, compost, peat) [6,7,8] or inorganic colloidal soil fractions (clay minerals–CM, oxides) [9,10,11] are believed to be decisive in many pesticides immobilization in soil. It has been postulated that in soils of low organic carbon content, clay minerals and hydr(oxides) may significantly contribute to the sorption of particular agrochemicals [12]. In addition to that, an important factor in determining the availability of potential sorption sites is soil pH, as it controls the form and thus reactivity of both the potential sorbate and various inorganic and organic soil colloids. Nevertheless, in studies of non-ionic organic compounds, the pH influence is often neglected, as these neutral substances are present in their molecular forms in the whole pH range. Meanwhile, pH modulates surface charges in soil, together with solubility of various soil colloids, and is, at the same time, a limiting factor in terms of the possible interaction types and sorption strength among the pollutant and soil constituents. 

According to many authors, uncharged chemicals are primarily bound to soil organic matter [13,14,15] and hydrophobic bonding is postulated as the main route of their uptake [14,16,17]. Several mechanisms have been proposed for the interaction of non-ionic chemicals with humic substances (HS) in soil [18,19], such as van der Waals forces, hydrogen bonding, ligand exchange and charge transfer complexes. They may operate during the interaction between a given pesticide and soil particles depending on the content of particular SOM fractions and the nature of the organo-mineral associations [5]. 

Recently, more attention has been paid to the composition of SOM in terms of its role as an indicator of landscape changes [20], soil physical quality [21], cultivation practices on arable lands [16,22], as well as predictors of pesticide affinity to soil [23]. Despite these paramount findings and their importance, by 2020, Novotny et al. [23] counted only a few papers referring to the relationship between the chemical structure of SOM and the organic contaminant sorption capacity of the whole soil. According to them, particular SOM fractions (e.g., humic acids, aromatic and aliphatic structures) were found to be useful proxies in understanding the interaction between SOM and pesticides [24,25,26,27]. However, to obtain a complete overview of the individual SOM fractions on the whole soil capacity towards organic chemicals, a joint approach is necessary. It should take into account not only the agrochemical affinity to individual SOM components but also the content and mutual share of particular organic carbon fractions of the soil, such as total extractable (TEC), humic acids (CHA), or residual carbon (CR) fractions. Information on the behavior of organic contaminants in soils related strictly to organic carbon (OC) composition and its individual fraction content is still limited, although it might be decisive in elucidating the soil capacity towards agrochemicals.

Non-ionic pesticides, namely carbaryl, carbofuran and metolachlor, were chosen for the study as their properties are intermediate between polar and non-polar compounds. They vary in water solubility, the type of functional groups present in their structure and hydrophobicity. Carbaryl and carbofuran belong to the carbamate insecticides group. Both compounds are non-persistent, moderately mobile in soil and of low (carbaryl) to moderate (carbofuran) water solubility, which strongly influences their environmental fate, including leaching potential [10,12,28,29]. Despite their suspected endocrine activity and disapproval for application in certain EU countries, they are still used in many parts of the world and detected in soils of some central and eastern European regions, including Poland [30]. Metolachlor is a selective pre-emergency herbicide [31], which belongs to the aniline derivatives group. Its moderate adsorption in soil is correlated with relatively high water solubility of the chemical [32], while its high lipophilicity places it in the group of substances suspected of endocrine activity [33], similar to carbaryl and carbofuran. 

Both carbamate and metolachlor retention in soil is attributed to its organic carbon content. Carbaryl sorption in soil is mostly connected to its SOM content, as well as illite (Il) and goethite (Ge), all of which have been shown to greatly increase pesticide retention when added to soils [10]. Studying model clay minerals and hydroxides, Chen et al. [9] discovered that carbaryl was efficiently adsorbed on montmorillonite (Mt), kaolinite (K) and goethite. Carbofuran sorption in soil was also related to organic matter and clay content [12], although Singh and Srivastava [29] claim that the mineral content of the soil is more important than that of OC when considering agrochemical retention. Indeed, in some other studies it was found that goethite and montmorillonite were the major soil constituents responsible for carbofuran retention in loamy sand soil of low organic carbon content [10]. Organic matter was also postulated as the predominant adsorbent for metolachlor, which was sufficiently mobile in soils low in organic carbon [34]. In the previous study it was found that the sorptive potential of soil towards the chemical was greatly enhanced by its amendment with montmorillonite, humic acids, goethite and illite [10]. Meanwhile, studies on the sorption of these two groups of non-ionic pesticides under varying soil pH conditions are scarce and often contradictory. Rama Krishna and Philip [35] reported that adsorption of neutral molecules increases with an increase in pH, which was corroborated by research on carbofuran in lateritic soils [12]. Metolachlor sorption was the most effective at a soil pH of five [36], whilst Mondal et al. [37] reported that carbaryl was efficiently retained in alluvial soils at a pH of six. In general, the majority of carbamates studies considering their adsorption capacity under various pHs of soils are contradictory and limited, as most of them are related to the chemical hydrolysis of the insecticides under neutral and alkaline pH conditions [38,39,40,41,42].

Considering their importance, this study was aimed at investigating the sorption of non-ionic pesticides with endocrine activity (carbaryl, carbofuran, metolachlor) on agricultural soils of different SOM fractional compositions and under various pH ranges. In order to comprehensively analyze the environmental behavior of a particular pesticide in soil, the study was performed on the whole soil material, not a single-component system. The evaluation was undertaken based on pesticide sorption-desorption studies performed on loamy sand and loam topsoil materials and an estimation of their release from the tested soils under a pH range of three to seven. Isothermal parameters obtained from Langmuir, Freundlich, Temkin and Dubinin–Radushkievich models were then related to individual SOM fraction contents and shares in the OC of the soils. The overall aim was to determine which SOM fraction represents the potential sink for the tested non-ionic agrochemicals in soils and to establish the pH conditions for their most effective retention. Such information may significantly contribute to the risk assessment of surface and groundwater contamination by the tested chemicals and fill the gaps in understanding of the attenuation phenomena of organic xenobiotic off-site migration in the environment. 

## 2. Results and Discussion

### 2.1. Sorption Isotherms and Their Mathematical Approximations

**Figure 1:**
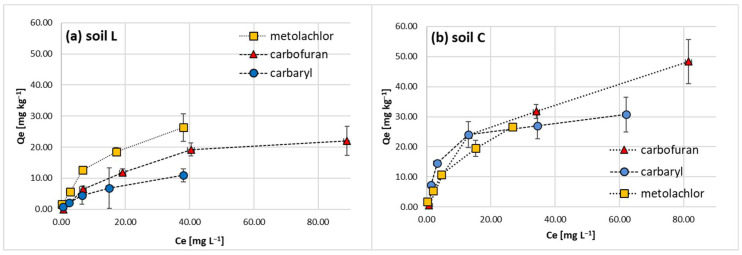
Adsorption isotherms of carbaryl, carbofuran and metolachlor on soil L (**a**) and soil C (**b**). Error bars represent standard deviation of triplicate samples.

**Figure 2:**
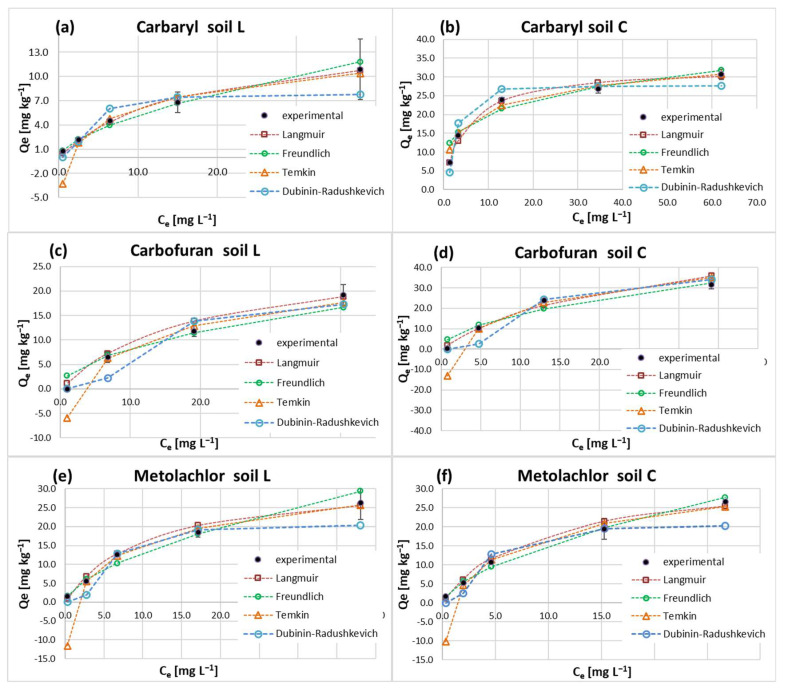
Comparison of the experimental data with the Langmuir, Freundlich, Temkin and Dubinin–Radushkevich isotherms for the adsorption of carbaryl (**a**,**b**), carbofuran (**c**,**d**) and metolachlor (**e**,**f**) in soils L and C.

Tested pesticide classes were subjected to sorption studies on sandy loam (L) and loam (C) topsoil materials and resulting isotherms (Figure 1) were approached with Langmuir, Freundlich Temkin and Dubinin–Radushkievich (D–R) models (Figure 2). Linear sorption parameters calculated for the obtained Langmuir-type [43] isotherms are summarized in Table 1. Applied models revealed high correlation coefficients (R^2^ > 0.950), except for the D–R model. The resulting L-shape of the isotherms is due to adsorption site limitations with the increasing pesticide concentration and presumably the energetic heterogeneity of the studied soils, as in the case of most solids [44]. In fact, the experimental isotherms approached a saturation upper limit but did not reach a complete plateau. This implies that at high adsorbate concentrations, weak intermolecular forces among the studied non-ionic pesticides might occur and participate in multilayer formation [45,46]. 

The maximum monolayer adsorption capacity Q_max_ (mg kg^−1^) of the Langmuir model, evaluated for the hydrophobic pesticides on soil L, correlated well with their Freundlich K_f_ constants and followed the increasing order of carbaryl (15.25) < carbofuran (27.93) < metolachlor (32.47). C soil Q_max_ parameters for carbaryl and carbofuran were doubled compared to L soil, whereas no significant change in case of metolachlor was noticed. 

Analogous trends to those of Q_max_ were observed on both studied soils for the theoretical monolayer sorption capacity (Q_m_) calculated with the D–R approach. It points to a significant increase in the sorption sites active in insecticide retention. In addition, the hydrophobic character and low water solubility of the studied carbamates may favor their diffusion within organic matter structures. According to the literature, diffusion and the following physical entrapment within the soil–organic matter matrix is a common sorption mechanism, especially in the case of pesticides independent of any traditional chemical adsorption mechanisms [47]. 

**Figure 3:**
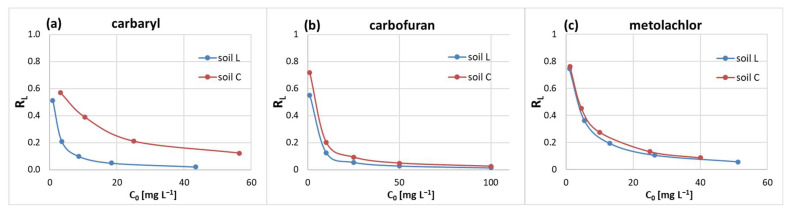
Variation of the separation factor (R_L_) in relation to initial concentration range of the studied (**a**) carbaryl, (**b**) carbofuran, (**c**) metolachlor on soils L and C.

Freundlich and Langmuir isotherm parameters, namely, the adsorption intensity (n_f_) and separation factor (R_L_), ranged from 0.248 to 0.611 (Table 1) and up to 0.80 (Figure 3), respectively. This indicates favorable (0.1 < n_f_ < 1.0 [48]) and reversible (0 < R_L_ < 1 [49]) adsorption of the solute and suggests that the pesticide molecules preferred high-energy sites followed by sorption at lower-energy sites [50,51]. 

The Temkin isotherm constant related to the heat of adsorption (b_T_), and the binding constant of the pesticide to a soil (A) exhibited decreasing and increasing trends, respectively, when comparing soils L and C (Table 1). According to the model assumptions, b_T_ would more often decrease than increase with increasing coverage [52]. Hence, it can be concluded that the lower values of the constant obtained for loamy soil indicate a higher degree of surface coverage with the pesticides compared to sandy loam soil material. Furthermore, from the b_T_ value, the nature of the process may be elucidated [53]. In the case of physical sorption, it should be lower than 4.184 kJ mol^−1^ (1 kcal mol^−1^), while for chemical sorption it is above 41,840 kJ mol^−1^ (10 kcal mol^−1^). Thus, the values of the b_T_ parameter (Table 1) suggest that the sorption of carbamates and metolachlor in L and C soils is of a physical nature. This was also confirmed by the E_a_ values obtained within D–R isotherms (Table 1), which were lower than 8 kJ mol^−1^ and may be partly due to the van der Waals interaction between the pesticides and the hydrophobic soil components.

Freundlich K_f_ parameters, which revealed the highest variation when comparing both studied soils, were used for the calculation of the standard Gibb’s free energy change (ΔG°) of the tested pesticides (Table 2). Prior to ΔG° calculations, K_f_ parameters were converted to the dimensionless standard equilibrium constants using the appropriate equations, as suggested in the literature [54,55,56].

The negative ΔG° values obtained for all studied pesticides on soils L and C point to the spontaneous character of the sorption. The significant increase and the highest negative ΔG° value obtained for carbaryl sorption in C soil indicates that in the case of this insecticide, the adsorption capacity would be higher for clay than for sandy soil, as for carbofuran (Table 2). Meanwhile, ΔG° of much similar magnitude was obtained for metolachlor sorption in both studied soils. The values of this thermodynamic parameter calculated for the studied pesticides are comparable to those found in the literature, although a limited number of papers performing ΔG° calculations using dimensionless K_c_ can still be found [55,57,58].

In addition, K_f_ parameters were used to calculate organic carbon partition coefficient (K_oc_) and groundwater ubiquity score (GUS, [59]) indexes, providing information about pesticide mobility and leachability, respectively (Table 3). All calculated K_oc_ values (except for carbaryl sorption in C soil) were below 500, which points to minimum or no adsorption of the chemicals into the soil and indicates high probability of their runoff or leaching [1,60]. A similar mobility ranking by FAO [61] based on log K_oc_ values classifies all of the pesticides as of moderate mobility, achieving log K_oc_ values in the range of two to three. Interestingly, despite the fact that the metolachlor sorption magnitude is higher in the loam than in the sandy loam soil under study, the K_oc_ value for herbicides in C soil is lower than in L soil. This might suggest that organic matter is not the main soil constituent responsible for metolachlor immobilization in soil. 

Calculated ground ubiquity score (GUS) indexes point to the following decreasing order of leaching threat in L soil: carbofuran > carbaryl > metolachlor and C soil: carbofuran > metolachlor > carbaryl. The lowest risk of soil C percolation (GUS value ≤ 1.8) was obtained for carbaryl and metolachlor and the chloroacetanilide pesticide in L soil. Carbofuran potential for movement toward groundwater was moderate in soil C (2.47), approaching high (> 2.80) in loamy sand soil. 

**Figure 4:**
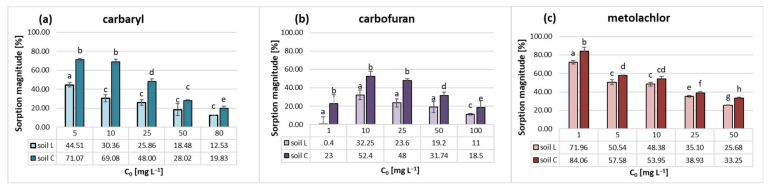
Sorption magnitude of carbaryl (**a**), carbofuran (**b**) and metolachlor (**c**) in soils L and C. Error bars represent standard deviation of triplicate samples. Different letters indicate significant differences (*p* < 0.05).

The retention of carbaryl, carbofuran and metolachlor on the investigated loamy sand and loam soil materials was evaluated by comparing their sorption-desorption magnitudes. Studied pesticide trends in L and C soils are presented in Figure 4. They vary in the magnitude of sorption on loam and sandy loam soil studied for a given hydrophobic agrochemical. Nevertheless, their common feature is a decline in sorption magnitude with the increase in initial concentration, which confirms the resulting L-type shape of isotherms (Figure 1). 

In general, carbaryl was adsorbed up to 44.51% and 71.07% in L and C soils, respectively (Figure 4a). Carbofuran was characterized by a similar pattern; however, its maximum sorption magnitude was 33.50% of pesticide initial dose introduced to soil L and 55.0% in the case of soil C (Figure 4b). Both carbamates revealed a great discrepancy in sorption magnitude among sandy loam and loam soils. On the contrary, metolachlor sorption in both soils was very similar, with the highest magnitude among the studied pesticides (84.06% and 71.96% for soils C and L, respectively, Figure 4c).

**Figure 5:**
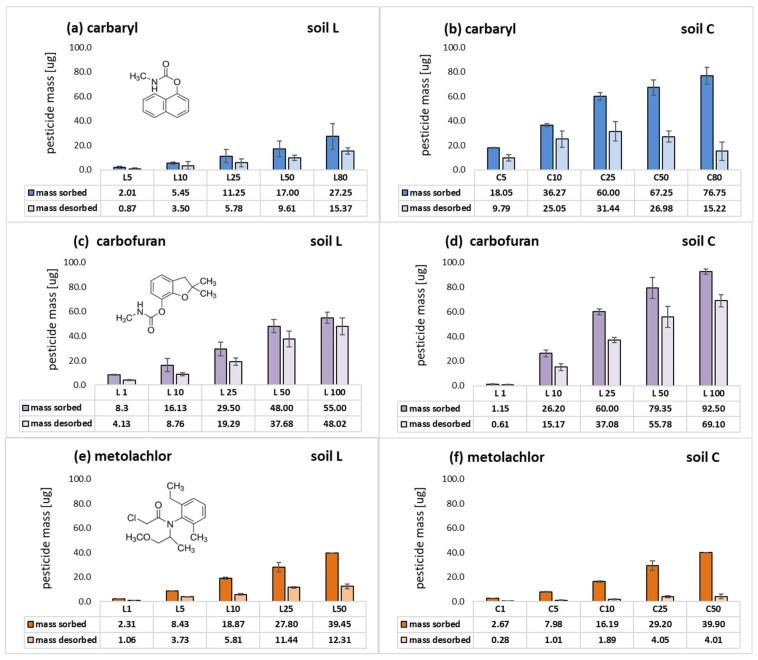
Comparison of carbaryl (**a**,**b**), carbofuran (**c**,**d**) and metolachlor (**e**,**f**) mass sorbed and desorbed in relation to different initial pesticide concentrations added to soils L and C. Error bars represent standard deviation of triplicate samples.

Meanwhile, desorption of carbamates and metolachlor achieved higher values in L rather than the C soil studied (Figure 5, expressed as a mass initially sorbed and desorbed). Carbaryl and carbofuran release from loamy sand soil was increasing along with the initial pesticide dose introduced to soil, achieving a maximum value of 62.6% and 87.0% of the pesticide dose initially sorbed, respectively.

Desorption in C soil was lower in the case of carbaryl (20.50–69.06% of the initial dose) and much higher for carbofuran in loam soil (from 49.76% of the lowest dose applied, up to approx. 74.70% in case of the highest initial concentration), making its sorption reversible [63] and enhancing a great threat of carbofuran percolation in soil. These results are consistent with the calculated GUS indexes (Table 3), confirming the low and slightly moderate potential of carbaryl to leach in soils C and L, respectively, and moderate potential for carbofuran in both studied soils. Metolachlor desorption in L soil was in a range of 31.20% to 45.89% and from 10.49% to 13.87% in C soil. This acetanilide pesticide release was the lowest among the group of the uncharged pesticides studied, as were the calculated GUS values (1.46 and 1.60 in soils L and C, respectively), suggesting its scarce leaching potential in both studied soils (Table 3). The strong adsorption and low desorption characteristics of metolachlor suggest that for the soils studied, the migration risk to groundwater should be lower than for the other uncharged pesticides examined.

In general, studied pesticides sorbed less to loamy sand than the loam soil (Figure 4), which contained more organic carbon and clay fractions.The highest negative standard Gibb’s free energy value obtained for carbaryl in soil C (Table 2) together with its lowest GUS index (1.12, Table 3) may indicate that the forces holding the insecticide in soil are relatively strong. Similarly, the relatively high and negative ΔG°, the lowest desorption magnitude (Figure 5e,f) and the highest log *p* value among the studied pesticides predestinate metolachlor to be efficiently retained in both soils. 

Taking into account the parameters approximated from sorption isotherms as well as the results of desorption studies, it can be concluded that the molecular interactions between the studied pesticides and the active soil constituents are of a physical nature. In general, this type of sorption for uncharged pesticides in soils is primarily governed by van der Waals forces, hydrophobic interactions and hydrogen bonding among the suitable, complementary functional groups of both interacting moieties [15,18,41]. 

### 2.2. Influence of Fractional SOM Composition on Carbaryl, Carbofuran and Metolachlor Retention in Studied Soils

According to the previous study on adsorption of the investigated uncharged pesticides in L and C soils amended with various soil colloids, agrochemical retention was attributed to their distribution in soil organic matter and inorganic colloids [10]. Both soils revealed similar mineralogical compositions according to which montmorillonite and kaolinite were major clay minerals. However, substantial variations were revealed in the fractional compositions of organic matter of soils L and C (Table 4). According to them, loamy sand material is rich in humic acid fractions (CHAs), which constitutes 40.07% of total organic carbon (TOC), whereas in loam soil residual carbon fractions (CRs, also called stable organic matter or humin (HN) fraction) represent 59.71% with a dominant share in TOC. This discrepancy in the mutual share of particular SOM fractions and their chemodiversity will have a direct impact on the magnitude and stability of interactions with pesticides [16]. Humic substances (HS) may react with uncharged pesticides via different modes, of which hydrophobic retention is regarded a common adsorption mechanism [18,64]. This may occur on hydrophobic active sites of HS where the competition for water is minimized [64]. These regions include aliphatic side-chains (common in humin fraction) or lipid constituents, as well as lignin-derived moieties with a high carbon content and a small number of polar groups. Van der Waals interactions also play an important role in the adsorption of non-ionic pesticides on complementary sites of HA. Since these forces are additive, their contribution increases with the size of the interacting molecule and with its capacity to adapt to the HA surface. This SOM fraction presumably consists of micelles of a polymeric nature, where the basic structure consists of an aromatic ring (di- or trihydroxy-phenyl type) bridged by -O-, -NH-, -N=, -S- and other functional groups that contain both free -OH and -COOH groups and the double linkages of quinones [18]. These functionalities may be involved in adsorption of non-ionic polar pesticides directly via hydrogen bonding or indirectly by affecting the hydrophilic/hydrophobic balance of the SOM surface [64]. Therefore, it is well documented that natural SOM fractions (humic and fulvic acids, humin), as well as different biopolymers (e.g., cellulose, lignin, cutin), due to the structural differences, may exhibit various affinities to organic contaminants, which can be also evaluated by their various K_oc_ coefficients [23].

When we compare the calculated K_oc_ values for carbamates in the investigated L and C soils (Table 3), an increase in the soil C affinity to the pesticides can be elucidated. This is particularly evident in the case of carbaryl, where an over 5.7 times higher K_oc_ value for loamy soil is obtained, whereas for carbofuran the increase is by a factor of 1.25. In addition, the maximum monolayer adsorption capacities Q_max_ of carbamates approximated for C soil were twice as much as for L soil, which correlates well to the doubled (compared to L soil) content of the residual carbon fraction of C soil (Table 4). Since the diverse share of OC fractions of the studied soils will evoke structural changes in SOM, this should be also reflected in their degree of sorption site heterogeneity [65]. According to a dual-mode sorption model by Xing and Pignatello [66], the more “glassy” or condensed nature of the organic matter, such as in the case of its humin (HN) or residual carbon (CR) fraction, the higher the nonlinearity of the sorption isotherm. Furthermore, a lower n_f_ value for carbaryl sorption in C soil, compared to sandy soil, points to a higher degree of sorption site heterogeneity of loam soil components active in insecticide uptake, which in turn may be due to its high CR fraction content. Since the pH of both tested soil materials was similar, the enhancement of C soil’s adsorption observed for the two carbamates can be related to its residual carbon and partly to humic acid carbon contents. This may be due to the pronounced hydrophobic retention of the carbamate by the CR fraction, supported by hydrogen bonding via the carbaryl’s amide carbonyl group and carboxylic groups of the HA fraction [67].

The postulated particularly high affinity of carbaryl to the soil residual carbon fraction is in agreement with the studies of Murthy and Raghu (1991, [68]), who found that nearly 78.9% of carbaryl was accumulated in the humin fraction of sandy loam soil, followed by the fulvic acid fraction (19.2%), whereas the least amount of pesticide was measured in the humic acid fraction (1.9%). Worth mentioning is the pH (6.8) of the investigated soil, which was very similar to that of the L soil studied herein. To further corroborate this finding, according to the literature, among the OC fractions the residual stable lignin fraction (of aromatic backbone, containing 64–65% of carbon) followed by CFA and CHA accounted for greater adsorption of some pesticides, including carbaryl [27]. In another study [41] it was also proven that carbaryl sorption on humic acid as a single sorbent was scarce in comparison to a more aromatic biochar, where a hydrophobic effect together with van der Waals forces were postulated to be responsible for insecticide effective retention, similar to the interactions claimed herein for soil C. Hence, the high CR content of C soil may significantly contribute to the generally higher sorption magnitude of carbaryl compared to sandy loam material. 

According to the literature [69], carbofuran predominantly incorporates into the recalcitrant humin fraction of SOM, constituting approximately 50% of the initial insecticide dose introduced into the soil. In a previous study [41], it was proven that retention of non-ionic carbamates on HA was much weaker (35.4% of carbofuran and 10.2% of carbaryl initial dose) than on more hydrophobic wheat-straw biochar. Therefore, hydrophobic attraction may also govern carbofuran uptake by the particular SOM fractions. In addition, studies performed on the same L and C soils investigated herein [10] have also shown that carbofuran retention was mainly controlled by goethite, expandable CMs, followed by HA (a slight but significant sorption increase) on sandy loam soil. This is in accordance with the study of Singh et al. [70], who found that carbofuran adsorption was strongly correlated with clay content followed by the organic matter content of soils. However, insecticide uptake on the loam soil material was attributed solely to montmorillonite, presumably due to the enhanced physical diffusion of the pesticide into Mt interlayers, governed by van der Waals forces [71]. It is commonly observed that the particulate organic (or organo-mineral) soil fractions form stable microaggregates where the retention of pesticides could depend more on steric factors and diffusion mechanisms than on the type and amounts of oxygen-containing functional groups [72]. Therefore, both organic (mainly CR) and inorganic (Mt) soil colloids contribute to carbofuran retention in the studied soils via hydrophobic bonding and van der Waals forces. However, care should be taken when assessing their mutual superiority and share in the insecticide uptake by soils. Nevertheless, the results obtained herein clearly demonstrate that carbofuran molecules were bound weakly to loam and sandy loam soils, confirming the physical nature of insecticide sorption and a great leaching potential. 

In the present study, metolachlor uptake by soil C was slightly but significantly higher than that of soil L (Figure 4c). Its affinity for organic carbon (K_oc_) of soil C (Table 3) decreased nearly 1.5 times in comparison to sandy soil. Taking that into account, it can be concluded that the higher herbicide K_f_ calculated for C soil (Table 1) indicates that apart from particular SOM fractions, other soil constituents must also contribute to its retention in loamy soil. This is in agreement with the studies of Weber et al. [73], who related metolachlor uptake by soil to its organic matter, humic matter (HM) and clay contents. In general, they postulated that the herbicide sorption by soil was of a physical nature and was based on the following mechanisms: hydrophobic bonding to lipophilic SOM sites, charge–transfer interactions, van der Waals forces and H-bonds on polar surfaces of clay minerals. It is in line with other studies where it was proven that metolachlor exhibits a great affinity for the HA polar functional groups, which renders the formation of H-bonding via the carbonyl site and for hydrophobic bonding sites such as aromatic rings [41], which may even preclude H-bonding [18,74]. The recent results of the herbicide sorption on Chernozem soil as well as its HA and HN fractions [75] corroborate the higher affinity of the herbicide for humic acids. Kozak et al. [76] studied the adsorption of metolachlor by various SOM constituents, including humic substances (HA and FA), humin and nonoxidizable soil organic matter. Based on the estimated partition coefficients normalized to organic carbon, they proved that fulvic and humic acids exhibited the highest affinity for the herbicide among the soil OC constituents. On the contrary, studies of Ding et al. [65] revealed that the humin fraction was superior to humic acids in metolachlor uptake. However, the HN fractions they isolated from soils were, uncommonly, much more aromatic than the HA, and the qualitative differences in their structural characteristics were correlated with the long-term tillage management of the soil. This finding is in contradiction with the latest studies on the nature of humin, which demonstrate that its major components are predominantly aliphatic hydrocarbon functionalities [77]. Nevertheless, it must be kept in mind that the type of extraction or isolation method will strongly influence the chemodiversity of this stable organic matter fraction [16,75,78], as well as the results of the studies conducted on the single-sorbent systems. Among the inorganic soil constituents, particularly clay minerals, bentonites and montmorillonites were found to be effective sorbents of metolachlor [79]. According to the literature, the herbicide sorption takes place dominantly as molecular species on hydrophobic microsites of Mt surfaces [8,80]. Presumably, metolachlor is adsorbed at the uncharged and hydrophobic siloxane groups of the clays silicate surface and held there by accepting hydrogen bonds from water molecules around the interlayer cations. Hence, in the present study, it can be postulated that in the case of C soil, the overall sorption magnitude of herbicide can be attributed in part to the humic acid fraction content (decreased by a factor of two in relation to L soil) and increased clay minerals content (mainly Mt [10]). 

### 2.3. pH-Dependent Sorption of Carbamates and Metolachlor on L and C Soils

**Figure 6:**
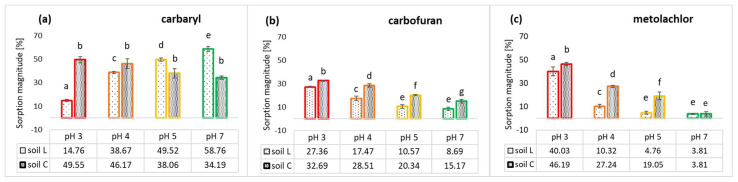
Sorption of non-ionic pesticides: (**a**) carbaryl, (**b**) carbofuran, (**c**) metolachlor, studied under a pH range of three to seven in L and C soils. Results are expressed as mean values ± standard deviation (n = 3). Different letters indicate significant differences (*p* < 0.05).

Sorption of carbaryl by soil L was noted to increase along with the soil pH, appreciably up to pH seven (Figure 6a). According to some studies, carbaryl sorption is due, in part, to site-specific interactions between carbamate functional group and exchangeable cations [27,81], e.g., via Ca^2+^ bridging. Thus, the trend observed for sandy loam material may be explained by the increasing competition between hydrogen and Ca^2+^ ions for active sites on the adsorbent surface with pH decline. In such case, available adsorption domains for carbaryl on clay minerals (kaolinite and montmorillonite) and SOM fractions (Table 4) on soil L will decrease. Furthermore, it was found that an increase in soil pH decreases the amount of extractable carbaryl residues [68], increasing bound residues at the same time, which can be retained by the CR fraction of soil OC. The sorption magnitude found for carbaryl on soil C is in a narrow range of 34.19% to 49.55% and seemingly declines with a pH increase, but the differences are not statistically significant (Figure 6a). The higher sorption magnitude observed for carbaryl on C soil of a pH of three to four compared to L soil is presumably related to its higher organic carbon content, as well as SOM composition (Table 4). Over the range of experimental pH values, some part of surface functional groups (e.g., carbonyl, hydroxyl, carboxyl) should become more protonated with a pH decrease. This results in less negatively charged SOM fractions abundant in hydrophilic moieties at a lower pH [82], which together with the high share of CR (59.71% of TOC) may favor adsorption of the 1-naphthyl-N-methyl moiety of carbaryl to the active sorption sites via hydrophobic bonding [28].

Meanwhile, desorption of the agrochemical from the two soils studied was the lowest at pH three; however, magnitude of the phenomena varied with the soil (Table 5). In loam, material carbaryl desorbed up to 25.34% (at pH 7) of the dose initially adsorbed, whereas in loamy sand soil its release ranged from 47.23% to 59.08%. Two conclusions can be drawn from these observations. Firstly, low pH reduces carbaryl desorption from loamy sand and loam soils studied, enhancing its retention. Secondly, carbaryl is more efficiently retained by loamy material, suggesting that the residual carbon fraction may be essential in the stabilization and immobilization of agrochemical residues in the soil environment. 

Carbofuran sorption exhibited an inverse trend with a pH increase for both studied soils (Figure 6b). Its sorption magnitude was significantly higher for loam soil material, achieving values of 27.36% and 32.69% of the carbofuran initial dose introduced for soils L and C, respectively. This discrepancy in the insecticide uptake by soils may be related to their different SOM quantities and compositions (Table 4), which significantly influence the number of specific potential sorption centers for the adsorbate. However, these results are in contradiction to the work of Rama Krishna and Philip [35], who found that with a pH increase from two to eight there was an increase in the sorption of carbofuran, methyl parathion and lindane in sandy, red, clayey and compost soils. A similar observation was found by Hsieh and Kao [12], who reported the enhanced adsorption of carbofuran in lateritic soils with a pH increase. Nevertheless, lateritic soils, rich in aluminum and iron minerals, are formed in wet, hot tropical areas, contrary to the soil materials of temperate climate and low inorganic colloids content investigated herein. Another study by Gupta et al. [82] revealed the inverse to pH adsorption trend for carbofuran, corroborating the results presented in this paper. Similarly, Salman et al. reported a favorable adsorption of carbofuran in acidic soils. Meanwhile, desorption of the insecticide was higher in sandy loam soil material (up to 98.1% of carbofuran initial dose at pH seven, Table 5), suggesting that the forces holding it there were weak, enhancing a serious threat of groundwater contamination. Carbofuran release in soil C was significantly lower (maximum of 33.05%), suggesting the high efficiency of soil colloids in its immobilization in soil.

Metolachlor sorption in soil L is also pH dependent, and similarly to carbofuran, it declines inversely with pH. The same behavior of pesticide can be observed in loamy soil (Figure 6c), which reveals an overall higher sorption capacity under the experimental conditions than the loamy sand soil material. This can be explained by the fact that a lower pH facilitates hydrogen bonds between the carbonyl oxygen of metolachlor and H atoms of carboxyl and hydroxyl groups of the organic moieties and charge–transfer (π-π) bonds between the aromatic nucleus of the herbicide and the aromatic rings on the organic matter surfaces [83]. Meanwhile, the desorption of agrochemicals (Table 5) increases with pH in sandy loam soil and is hindered by lower pH (three to five) conditions in case of loam soil material. Nevertheless, its magnitude is much higher in L soil (61.67%), whereas it seems to be limited by some C soil constituents (maximum desorption of 18.84% at pH seven), as they retain agrochemicals more efficiently than loamy sand.

## 3. Materials and Methods

### 3.1. Chemicals

Pesticides chosen for this study were carbaryl (1-naphthyl methylcarbamate), carbofuran (2,2-Dimethyl-2,3-dihydro-1-benzofuran-7-yl methylcarbamate) and metolachlor ((*RS*)-2-Chloro-*N*-(2-ethyl-6-methyl-phenyl)-*N*-(1-methoxypropan-2-yl)acetamide). All the active agrochemicals substances were purchased from Merck (Darmstadt, Germany) with a reported purity of >98%. In the case of metolachlor, its stock standard solution in acetonitrile (100 mg L^−1^) was used (HPLC grade). All reagents were of analytical grade. Working standard mixed solutions were prepared weakly by diluting each individual stock solution in 10 mM CaCl_2_ prepared in Milli-Q water and stored at 4 °C. The pesticides studied and some of their physical–chemical properties are presented in Table 6.

### 3.2. Arable Soils under Study

The representative 2 topsoil samples (0–30 cm) from farmlands in Ligota Piękna (near Wrocław, Poland) were randomly collected for the study (at 12 points within each location). They were selected due to the agricultural land use and texture, as sandy loam (L soil material) represents 13.3% and loam (C soil) represents 16.8% of the topsoil in south-west Poland [84]. They are the 2 most abundant groups among light- and heavy-textured soils of the region. According to the WRB classification [85], the soils were mostly Phaeozems and Umbrisols. Prior to the analyses, soil materials were air-dried, crushed gently and sieved through a sieve with a mesh size of 2 mm. The fraction of the soils <2 mm was collected and used in the study. Table 7 shows a summary of the physicochemical properties of the investigated soil materials and was derived from a previous study [10]. 

In the fraction <2 µm of both soils, montmorillonite was the major clay mineral, accompanied by kaolinite and small amounts of highly dispersed quartz. In addition, both soils contained low level of carbonates at 0.6% and 1.1% in L and C soil, respectively. Ca^2+^ was the main exchangeable cation in both tested soils.

#### 3.2.1. Chemical Fractionation of the Humic Material

In order to correlate the soils affinity for the studied pesticides to a particular soil organic matter fraction, the extraction and chemical fractionation of the humic substances from investigated L and C soils was conducted. It was undertaken according to the method recommended by the International Humic Substances Society (IHSS), described in Swift [86], with some modification [87,88,89,90]. The first step in the procedure was sample decalcitation with H_2_SO_4_ [91] in order to obtain a low-molecular-weight fulvic fraction (CAC). In the next step, soil was exhaustively extracted with 0.1M NaOH until the supernatant was completely discolored. As a result, a fraction called total extractable carbon (TEC) was obtained. It consisted of humic acids (HA) and fulvic acids (FA). In the subsequent step, the alkaline extract was acidified to pH 2 to precipitate the humic acid fraction (CHA), which was then centrifuged from the CFA fraction remaining in the solution.

The carbon of humic acid (CHA) content was calculated from the difference between total extractable carbon (TEC) measured in alkali (NaOH) extract and carbon of fulvic acid (CFA) fraction after HA precipitation at a pH of less than 2. Carbon of nonhydrolyzable humic substances (also called residual carbon or humin fraction, CR) was calculated from the difference between total organic carbon (TOC) and sum of humic and fulvic acid fractions (CR = TOC − (CHA + CFA)).

Total organic carbon and concentrations of organic carbon in the subsequent fractions were measured after each extraction step on an Enviro TOC/TN analyzer (Elementar, Langenselbold, Germany).

#### 3.2.2. Preparation of L and C Soils of Various pH Levels

To set a series of soil L and C at various pHs in the range of 3–7, the following procedure was implemented. An aliquot (200 g) of each soil (L or C) was placed in a beaker, soaked with 50 mL of deionized water and stirred for 12 h. Small volumes of 1 M HCl solution were added dropwise to a soil slurry and stirred to obtain the desired final pH values. The samples were equilibrated for another 12h followed by the pH measurement. The procedure was repeated until each soil series contained samples of pH adjusted to approximately: 3, 4, 5 and 7. Obtained soils were air-dried, ground and subjected to the final pH test. Each soil sample (n = 3) was equilibrated with 10 mM CaCl_2_ solution in a ratio of 1:2 (used in the batch studies) to ascertain that the experimental pH values would be maintained. 

The pH range was set to cover the values typical for undisturbed soils, excluding values higher than 7. This was due to the fact that investigated carbamates undergo hydrolysis in alkaline conditions [38,42] and, furthermore, in pH values exceeding 7, humic acid fraction occurs in its dissolved form. Therefore, to obtain reliable results concerning carbamate concentration in a solution after sorption and to avoid the loss of potential sorbent from the solid phase, the upper limit for the pH range investigated was set to 7. 

### 3.3. Batch Equilibrium Sorption Studies

OECD 106 and USEPA guidelines for the testing of chemicals were adopted for the sorption-desorption studies [63]. They were conducted separately for the sandy loam (L) and loam (C) soils with each of the 3 pesticides tested. Performing batch studies on whole soils allowed for the preservation of complex chemical compositions and mutual interactions among constituents of soil organic matter and inorganic colloidal fractions, as well as their natural chemodiversity. The preliminary investigation showed a soil to solution (10 mM CaCl_2_) ratio of 1:2 was the most appropriate for all samples [10]. Therefore, subsequent experiments were carried out using this ratio.

Triplicate soil aliquots (5 g of air-dried soil L or C) were equilibrated with 10 mL of each pesticide aqueous solution by gently rotating in an end-over shaker at 20 ± 2 °C for 24 h [10]. The concentration ranges for the batch equilibrium studies were 0–100 mg L^−1^ for carbofuran, 0–80 mg L^−1^ for carbaryl and 0–50 mg L^−1^ for metolachlor on both studied soils. After equilibration, the soil suspensions were centrifuged for 15 min at 10,000 rpm. The concentration of the tested pesticide in the supernatant (C_e_; mg L^−1^) was measured, and the amount of pesticide sorbed on the soils (Q_s_; mg kg^−1^) was calculated from the concentration difference between the initial pesticide solution (C_0_) and C_e_:

Q_s_ = [(C_0_ − C_e_)V]/m, (1)

where V is the volume of solution (L) and m is the mass of soil (kg).

To evaluate the influence of pH on the selected carbamates and metolachlor adsorption in L and C soils, a single-concentration sorption-desorption experiment [63] was performed. The concentrations of the tested pesticides were as follows: carbaryl—10 mg L^−1^ and 20 mg L^−1^ (for L and C soils, respectively); carbofuran—15 mg L^−1^ and metolachlor 10 mg L^−1^ for both studied soils. Single point concentrations were chosen based on the batch studies, as they represent the pesticide initial concentration for which the sorption magnitude was maintained above 40% [63]. The soil to solution ratio, equilibration time, measuring conditions and methods used to calculate the sorption magnitude were identical to those in the batch experiment described above. Desorption studies were performed straight after taking up the supernatant after 24 h of equilibration. To the same vessel with the soil, an aliquot of background (10 mM CaCl_2_) solution was added and stirred for another 24 h. After centrifugation, the supernatants were filtered (0.45 μm) and subjected to quantitative analysis with LC/MS-MS (Thermo Scientific TSQ Quantum Access MAX) [41]. Briefly, 10 μL of the sample was injected into the analytical column (Thermo Scientific Hypersil GOLD column, 50 mm × 2.1 mm, with 1.9 μm particle size; flow rate of 0.50 mL min^−1^). Deionized water with 0.1% of formic acid constituted the mobile phase A, and 90% acetonitrile, 10% deionized water and 0.1% of formic acid was the mobile phase B. Gradient elution was applied as follows: 100% of eluent A from the first minute, which was replaced linearly up to 100% by eluent B in 20 min. This composition was maintained for a further 3 min before 100% of eluent A was applied, followed by a re-equilibration time of 7 min to give a total run time of 30 min. The mass spectrometer was run in single reaction monitoring (SRM) mode. Argon (99.995%) with a pressure of 1.87 × 10^−3^ mbar was applied in the collision cell. A capillary voltage of 4 kV was used in positive ionization mode. The ion transfer capillary temperature was set to 380 °C and the vaporizer of the ESI probe to 400 °C.

Desorption (D) was calculated using the following formula:

D (%) = (Q_de_/Q_ad_) × 100%, (2)

where Q_de_ is the quantity of pesticide that desorbed (mg kg^−1^) and Q_ad_ is the quantity adsorbed after pesticide addition to the soil [92]. The desorption magnitude was expressed in relation to the pesticide amount initially sorbed during the first 24 h of contact with L or C soil [6].

### 3.4. Mathematical Modeling of Sorption Isotherms

From the batch studies performed, isotherms of an adsorption process were constructed using the Langmuir, Freundlich, Temkin and Dubinin–Radushkievich (D–R) models. They describe the distribution of adsorbate molecules between the liquid and the solid phase [93], showing a relationship between the amounts of pesticide adsorbed per unit weight of the soil (Q_s_) and the pesticide concentration in the solution at equilibrium (C_e_). The adsorption curves and values of important variables of the linearized forms of isotherm equations for the studied pesticides in the soil samples were also determined.

#### 3.4.1. Langmuir Model

This model determines the saturation of the available adsorption sites and assumes that the adsorbate can form a monolayer of molecules interacting with homogeneous adsorption sites of equivalent energies on the adsorbent surface and that no interactions between adsorbed species occur [1,94]. The retained amount increases until a limited value corresponding to the surface coating by a monolayer is reached. 

The expression for the model is given by the following equation:

Q_e_ = (Q_max_ K_L_C_e_/1 + K_L_C_e_), (3)

Q_e_ is the amount of adsorbate at equilibrium (mg kg^−1^); C_e_ is the equilibrium concentration of the adsorbate (mg L^−1^); Q_max_ is the maximum adsorption capacity (mg kg^−1^); K_L_ is the Langmuir constant (the binding energy associated with adsorbent and adsorbate, L mg^−1^).

The linearized form of the Langmuir equation derived on the basis of Equation (3) is expressed as:

C_e_/Q_e_ = 1/(K_L_Q_max_) + C_e_/Q_max_, (4)

Additionally, the Langmuir parameter (R_L_), also called a separation factor, was determined for the studied initial concentration range:

R_L_=1/(1+ K_L_C_0_), (5)

Adsorption can be classified as irreversible if R_L_ = 0; favorable if 0 < R_L_ < 1; linear if R_L_ = 1; or unfavorable if R_L_ > 1, informing about the change in soil adsorption strength, the type of isotherm and adsorption state [95,96].

#### 3.4.2. Freundlich Model

The Freundlich isotherm is an empirical model describing multilayer adsorption with non-uniform distribution of adsorption enthalpy and affinities onto the heterogeneous adsorbent surface without lateral interaction [92,97]. The energetically favored binding sites are theorized to be occupied first and the binding strength decreases sequentially with increased coverage of the sites. The model can be described by the following equation:

Q_e_ = K_f_ C_e_^nf^, (6)

where Q_e_ is the amount of adsorbate at equilibrium (mg kg^−1^); C_e_ is the equilibrium concentration of the adsorbate (mg L^−1^) and K_f_ is the Freundlich affinity coefficient or Freundlich solid-water distribution coefficient (mg ^(1-n)^ L^n^ kg^−1^). K_f_ represents the pesticide affinity for soil, with high values indicating a stronger adsorption for the pesticide and also suggesting a lower mobility of the substance in the soil profile [98,99]. n_f_ is the exponential coefficient (constant) associated with the energy distribution of the adsorption site, also being a measure of favorability of sorption (for the values of n_f_ between 0 and 1) [100].

The pesticide concentration in the soil and soil solutions are fitted to the linearized Freundlich sorption isotherm equation:

ln Q_e_ = ln K_f_ + n_f_ ln C_e_, (7)

The Freundlich equation in a linear mode yields a linear plot in which the slope is n_f_ and characterizes the isotherm nonlinearity.

Based on the constants obtained from the Freundlich approximation of the experimental data, a thermodynamic parameter determining the spontaneity of the adsorbate–adsorbent interactions, namely, standard Gibb’s free energy (ΔG°), can be calculated:

ΔG°= −RT ln K_c_, (8)

where T is the temperature (K), R is the gas constant (8.314 J mol^−1^ K^−1^) and K_c_ is the standard equilibrium constant (dimensionless), which can be obtained by K_f_ conversion using the proposed equation [54,55,56]:

(9) $$K_{c}=\frac{K_{f}\;\rho }{1000}\;{\left(\frac{10^{6}}{\rho }\right)}^{\left(1-n_{f}\right)},$$

where ρ is the density of pure water (1.0 g/mL). For other experimental equilibrium constants K_L_, K_f_ and K_D_, the strategies for converting them into K_c_ were adapted [101,102,103,104] and must not be omitted when elaborating ΔG° calculations.

A negative ΔG° value indicates that the process is thermodynamically favorable and can conduct spontaneously. The greater the absolute magnitude of the ΔG° value, the higher is the extent to which the adsorption reaction may take place [35].

Additionally, based on the obtained K_f_ parameters for each pesticide, the organic-carbon normalized Freundlich distribution coefficients K_foc_ (in the text indicated as K_oc_—organic carbon-water partition coefficient) were calculated according to the equation below:

K_foc_ = (K_f_/OC) × 100%, (10)

where OC is the organic carbon content of the soil (%).

The organic carbon partition coefficient (K_oc_) indicates a magnitude of pesticide affinity for the organic fraction of the soil [105]. Its high values suggest that the pesticide is firmly fixed in the soil organic matter fraction, so only a small amount of the compound could undergo runoff to surface waters or leaching to aquifers. 

Pesticide leachability (or mobility) in the soil profile and the risk of groundwater contamination can also be calculated by determining the groundwater ubiquity score (GUS) index [59]:

GUS _index_ = (log t_1/2_) × (4 − log(K_oc_)), (11)

where t _1/2_ is the half-life of pesticide and K_oc_ is the distribution coefficient normalized to the organic carbon content of soil. GUS index values below 1.8 characterize non-leachers. 

#### 3.4.3. Temkin Model

The Temkin isotherm assumes a linear rather than logarithmic decrease in heat of adsorption of all molecules in the layer, while ignoring extremely low and very high concentrations of solute [92]. It also assumes uniform distribution of bounding energy up to some maximum. It is expressed by the following equation:

Q_e_= (RT/b_T_) ln C_e_ + (RT/ b_T_) ln A, (12)

Q_e_ = B ln C_e_ + B ln A, (13)

where Q_e_ is the amount of chemical adsorbed at equilibrium (mg kg^−1^) and C_e_ is the concentration of adsorbate in the solution at equilibrium (mg L^−1^). B is a constant defined by the expression B = RT/b_T_, b_T_ is the Temkin constant related to the heat of adsorption (J mol^−1^), T is the absolute temperature (K), R is the gas constant (8.314 J mol^−1^ K^−1^) and A is the Temkin isotherm binding constant (L kg^−1^). From the plot of Q_e_ vs. ln C_e_ (Equation (12)), B and A can be calculated from the slopes (B) and intercepts (B lnA), respectively.

#### 3.4.4. Dubinin–Radushkievich Model

The equilibrium data can also be applied to the Dubinin–Radushkievich (D–R) model to determine whether the sorption is of physical or chemical type. It is an empirical adsorption model that is generally used to express adsorption mechanisms with Gaussian energy distribution onto heterogeneous surfaces [106,107]. The linear form of the D–R isotherm is presented in the form of the following equation:

lnQe = ln(Qm) − βε^2^, (14)

where Qe is the amount of pesticide adsorbed into the soil (mg kg^−1^ ), Q_m_ is the theoretical monolayer sorption capacity (mg kg^−1^ ), β is the constant (mol^2^ J ^−2^), which is related to the average energy of sorption per mole of the sorbate as it is transferred to the surface of the solid from an infinite distance in the solution, and ε is the Polanyi potential, which is described by Equation (15), where T is the solution temperature (K) and R is the gas constant.

ε = RT ln(1 + 1/C_e_), (15)

The value of mean sorption energy, E_a_ (kJ mol^−1^), can be calculated from D–R parameter β, (obtained from the relationship of ln Qe and ε^2^, Equation (14)), according to the equation:

E_a_= 1/(2 β)^1/2^ (16)

The value of the mean sorption energy provides information about chemical and physical sorption. The E_a_ value ranges from approximately 1 kJ mol^−1^ to 8 kJ mol^−1^ for physical sorption and from 8 kJ mol^−1^ to 16 kJ mol^−1^ for chemical sorption [53].

### 3.5. Statistical Analysis

Data are expressed as the mean values of 3 replicates ± standard deviation (SD). The values of pesticide sorption and desorption magnitudes on L and C soils, including pH influence (both sorption-desorption data), were statistically compared using Student’s *t*-test (*p* < 0.05), prior to which the Shapiro–Wilk normality test was performed. The data were compiled using Microsoft Excel 2019 and GraphPad Prism 9.5.1.733 software.

## 4. Conclusions

Carbaryl, carbofuran and metolachlor sorption were better retained by loam than sandy loam topsoil material. Langmuir, Freundlich and Temkin models fitted well to the obtained experimental L-type isotherms. Approximated parameters point to favorable and spontaneous multilayer adsorption with a higher degree of surface coverage with the pesticides on C soil compared to soil L. The Dubinin–Radushkievich approach confirmed the physical nature of the studied agrochemical adsorption. GUS indexes indicate low leaching potential of metolachlor and moderate potential of carbofuran on both soils. Carbaryl is a non-leacher in loam, while on sandy soil it reveals a moderate percolation ability. 

Sandy loam material was more abundant in humic acid (CHA) fractions, whereas loam soil was abundant in residual carbon (CR). These differences in SOM composition were postulated to strongly influence the studied pesticide retention in soils. The residual carbon fraction of SOM is presumably superior in immobilization of carbaryl in the soil environment. Carbofuran retention in both soils was related to clay minerals (montmorillonite) as well as CR fraction content. The joint effect of humic acids and Mt was responsible for metolachlor uptake in both soils. Lower pH enhanced non-ionic pesticide retention in studied soils, except for carbaryl sorption in sandy soil. Therefore, SOM fractional composition and the mutual share of individual OC fractions together with pH are crucial for predicting the behavior of non-ionic pesticides in the soil environment.

## Figures and Tables

**Table 1:** Linear sorption isotherm parameters of carbaryl, carbofuran and metolachlor, approximated from the results of batch experiments on L and C soil.

Soil	Pesticide	Langmuir			Freundlich			Temkin			Dubinin–Radushkevich		
		Q_e_ = (Q_max_ K_L_C_e_/1 + K_L_C_e_)			Q_e_ = K_f_ C_e_^nf^			Q_e_ = (RT/b_T_)ln C_e_ + (RT/b_T_) lnA			Q_e_ = Q_m_ exp(−βɛ^2^)		
		Q_max_	K_L_	R^2^	K_f_	n_f_	R^2^	b_T_	A	R^2^	Q_m_	E_a_	R^2^
L	Carbaryl	15.25	0.06	0.988	1.27	0.611	0.994	0.784	0.70	0.978	7.82	0.500	0.841
	Carbofuran	27.93	0.04	0.992	2.66	0.496	0.959	0.390	0.40	0.973	18.43	0.236	0.861
	Metolachlor	32.47	0.10	0.964	3.29	0.602	0.989	0.322	0.74	0.994	20.72	0.500	0.909
C	Carbaryl	32.57	0.20	0.997	11.42	0.248	0.951	0.467	5.37	0.977	27.67	0.707	0.937
	Carbofuran	61.35	0.04	0.978	5.28	0.515	0.956	0.194	0.46	0.977	36.31	0.289	0.883
	Metolachlor	33.78	0.11	0.954	3.75	0.609	0.995	0.313	0.91	0.983	20.54	0.707	0.880

Q_e_–amount of adsorbate at equilibrium (mg kg^−1^); C_e_–equilibrium concentration of the adsorbate (mg L^−1^); Q_max_–maximum adsorption capacity (mg kg^−1^); K_L_–Langmuir constant (L mg^−1^); K_f_–Freundlich affinity coefficient (mg ^(1-n)^ L^n^ kg^−1^); n_f_–exponential coefficient associated with the isotherm nonlinearity; R–gas constant, 8.31 (J mol^−1^ K^−1^); T–the absolute temperature (K); b_T_–Temkin isotherm constant related to the heat of adsorption (kJ mol^−1^); A–equilibrium binding constant (L kg^−1^); Q_m_–theoretical monolayer sorption capacity (mg g^−1^); β–constant of the sorption energy (mol^2^ J ^−2^); ε–Polanyi potential; E_a_–mean sorption energy (kJ mol^−1^).

**Table 2:** Standard Gibb’s free energy change in tested pesticides on soils L and C.

Soil/Pesticide	Carbaryl	ΔG° [kJ·mol^−1^] Carbofuran	Metolachlor
loamy sand	−8.33	−14.10	−20.95
loam	−35.02	−23.97	−19.90

**Table 3:** Leaching indexes calculated for the studied pesticides in soils L and C.

Pesticide	L Soil				C Soil			
	K_f_	K_oc_	log K_oc_	GUS *	K_f_	K_oc_	log K_oc_	GUS *
carbaryl	1.27	134.15	2.13	1.87	11.42	761.51	2.88	1.12
carbofuran	2.66	280.00	2.45	2.64	5.28	351.87	2.55	2.47
metolachlor	3.29	346.32	2.54	1.46	3.75	250.00	2.40	1.60

* GUS-groundwater ubiquity score t ½ was taken from [62].

**Table 4:** Content of carbon fractions in the investigated loamy sand and loam soils, expressed as % of total organic carbon.

Soil	TOC	CAC	TEC	CHA	CFA	CR
	g 100 g^−1^			% of TOC		
L	0.95	6.87	61.76	40.07	21.69	31.37
C	1.50	4.64	35.65	20.17	15.47	59.71

TOC-total organic carbon, CAC-carbon of low-molecular fulvic fraction, TEC-total extractable carbon consisting of humic (CHA) and fulvic acids (CFA), CR-carbon of humin fraction.

**Table 5:** Desorption of non-ionic pesticides studied under a pH range of three to seven in L and C soils. Results are expressed as mean values ± standard deviation (n = 3). Different letters indicate significant differences at *p* ≤ 0.05.

Desorption at:	Carbaryl		Carbofuran		Metolachlor	
	Soil L	Soil C	Soil L	Soil C	Soil L	Soil C
pH 3	47.23 ^a^ ± 1.85	7.26 ^b^ ± 0.7	26.36 ^a^ ± 1.61	10.23 ^b^ ± 0.20	20.75 ^a^ ± 1.75	1.40 ^b^ ± 0.05
pH 4	51.98 ^c^ ± 1.08	21.92 ^e^ ± 0.67	55.86 ^c^ ± 0.29	12.29 ^d^ ± 0.24	49.77 ^c^ ± 2.76	1.62 ^b^ ± 0.57
pH 5	59.08 ^d^ ± 2.79	23.56 ^e^ ± 1.67	85.11 ^e^ ± 1.05	24.07 ^f^ ± 1.17	56.35 ^d^ ± 2.37	5.03 ^b^ ± 0.49
pH 7	56.03 ^d^ ± 0.38	25.34 ^e^ ± 1.35	98.01 ^g^ ± 1.90	33.05 ^h^ ± 0.49	61.67 ^d^ ± 6.73	18.84 ^e^ ± 0.14

**Table 6:** Basic properties of pesticides under study ^1^.

Property/pesticide	Carbaryl	Carbofuran	Metolachlor
Chemical structure	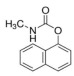	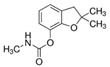	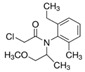
Water solubility (mg L^−1^) at 20 °C	9.1	322	530
Octanol-water partition coefficient, Log *P*	2.36	1.8	3.4

^1^ Data from PubChem open chemistry database and PPDB: Pesticide Properties Database, University of Hertfordshire.

**Table 7:** Basic physicochemical properties of tested arable L and C soils ^1^.

Soil	pH	C_tot_	C_org_	N	C_org_:N	CEC	Sand	Silt	Clay	Soil Texture
	(KCl)	%				cmolc·kg^−1^	%			
L	7.2	1.3	0.95	0.1	7.1	26.4	76	17	7	Sandy Loam
C	7.4	1.6	1.5	0.2	8.4	42.3	47	34	19	Loam

^1^ Previously published by Ćwieląg-Piasecka et al. [10].

## Data Availability

Data will be made available upon request.
